# Supplementary material for: Biosynthesis of ansamitocin P-3 incurs stress on the producing strain Actinosynnema pretiosum at multiple targets
Source: Commun Biol. 2023 Aug 18;6:860. doi: 10.1038/s42003-023-05227-w (PMC10439133; doi:10.1038/s42003-023-05227-w)
Supplement: Supplementary file 2 — Supplementary Information [file 42003_2023_5227_MOESM2_ESM.pdf]

Supplementary information

Biosynthesis of ansamitocin P-3 incurs stress on the producing strain *Actinosynnema pretiosum* at multiple targets

Qungang Huang[1,2], Xin Zhang[1,2], Ziyue Guo[1,2], Xinnan Fu[1,2], Yilei Zhao[1,2], Qianjin Kang[1,2], Linquan Bai[1,2]**

[1]State Key Laboratory of Microbial Metabolism, Shanghai-Islamabad-Belgrade Joint Innovation Center on Antibacterial Resistances, School of Life Sciences and Biotechnology, Shanghai Jiao Tong University, Shanghai 200240, China.

[2]Joint International Research Laboratory of Metabolic and Developmental Sciences, Shanghai Jiao Tong University, Shanghai 200240, China

*These authors jointly supervised this work. E-mails:

Linquan Bai, bailq@sjtu.edu.cn

Qianjin Kang, qjkang@sjtu.edu.cn

Table of Contents

SUPPLEMENTARY TABLES **5**

Supplementary Table 1. Strains used in this study and their properties and sources 5

Supplementary Table 2. Plasmids used in this study and their properties and sources 6

Supplementary Table 3. Primers used in strain constructions 8

Supplementary Table 4. Medium components 10

Supplementary Table 5. Target proteins identified from the chemoproteomic assay 11

Supplementary Table 6. Binding constants of target proteins with ansamitocin P-3 12

Supplementary Table 7. 95% confidence interval of k_{cat}, K_m and K_I of dTGD 13

Supplementary Table 8. 95% confidence interval of k_{cat}, K_m and K_I of FDTS 14

Supplementary Table 9. 95% confidence interval of k_{cat}, K_m and K_I of ALDH 15

SUPPLEMENTARY FIGURES **16**

Supplementary Figure 1. Construction and verification of the *asmA* deletion mutant HQG-3. 16

Supplementary Figure 2. Construction and verification of *asm7* deletion mutant HQG-1. 17

Supplementary Figure 3. Mass spectrometry analysis of QG-YNE. 18

Supplementary Figure 4. ^{1}H-NMR spectrum for QG-YNE 19

Supplementary Figure 5. ^{13}C-NMR spectrum for QG-YNE. 20

Supplementary Figure 6. Design of the photoaffinity binding protein profiling experiments to identify the ansamitocin P-3 binding protein spectrum in ATCC 31280 by proteomic analysis. 21

Supplementary Figure 7. Construction and verification of the *APASM_1052* overexpression strain. 23

Supplementary Figure 8. Verification of overexpression strains by gel

electrophoresis of PCR products. 24

Supplementary Figure 9. Amino acid sequence alignment revealed that dTGD in ATCC 31280 participates in dTDP-L-rhamnose biosynthesis. 25

Supplementary Figure 10. In vitro reaction for dTGD with dTDP-D-glucose and NAD+ as substrates. 26

Supplementary Figure 11. Overexpression of recombinant His-tagged target proteins in *E. coli* BL21(DE3) and analysis by SDS-PAGE. 27

Supplementary Figure 12. QG-YNE specific binding at 314-DNRDWWEPLKQR-325 of dTGD. 28

Supplementary Figure 13. Structure alignment of homologous proteins for dTGD and published crystal structure. 29

Supplementary Figure 14. Interaction of dTGD and AP-3 revealed by docking analysis..30

Supplementary Figure 15. Standard curve for dTDP-6-deoxy-D-*xylo*-4-hexulose at A_{320}. Calibration equation: $y = 0.9232\ x$, $R^2 = 0.9974$...................................32

Supplementary Figure 16. Thymidylate synthase and NADPH oxidase activities of FDTS. 33

Supplementary Figure 17. QG-YNE specific binding at 90-HFSYSQLSQR-99 of FDTS. 34

Supplementary Figure 18. Structure alignment of homologous proteins for FDTS and published crystal structure. 35

Supplementary Figure 19. Interaction of FDTS and AP-3 revealed by docking analysis..36

Supplementary Figure 20. Standard curve for NADPH at A_{340}. Calibration equation: $y = 0.8404\ x + 0.0018$, $R^2 = 0.9999$. 38

Supplementary Figure 21. Quantitative analysis of dTMP.................................39

Supplementary Figure 22. QG-YNE specific binding at 18-SRYDHFIGGEFTAPAK-33 of ALDH. 40

Supplementary Figure 23. Structure alignment of homologous proteins for ALDH and published crystal structure. 41

Supplementary Figure 24. Interaction of ALDH and AP-3 revealed by docking analysis..42
Supplementary Figure 25. Detection of ALDH activity by NADH generation. 44
Supplementary Figure 26. Quantitative analysis of acetyl-CoA. 45
Supplementary Figure 27. Quantitative analysis of citric acid and isocitric acid. 46
Supplementary Figure 28. Standard curve for AP-3. 48
SUPPLEMENTARY REFERENCES 49

SUPPLEMENTARY TABLES

Supplementary Table 1. Strains used in this study and their properties and sources

Strains	Features	Sources
Escherichia coli		
DH10B	F^- *mcrA* Δ(mmr-*hsdRMS-mcrBC*) φ80*lacZ*ΔM15 Δ*lac*X74 *rec*A1 *end*A1 *ara*D139 Δ(*ara*, *leu*)7997 *gal*E15 *gal*K λ-*rsp*L *nup*G	GIBCO BRL
ET12567(pUZ8002)	F^-*dam*-13::Tn9 *dcm*-6 *hsd*M *hsd*R zjj-202::Tn10 *rec*F143 *gal*K2 *gal*T22 *ara*-14 *lac*Y1 *xyl*-5 *leu*B6 *thi*-1 *ton*A31 *rps*L136 *his*G4 tsx-78 *mtl*-1 *gln*V44 Cml^R Kan^R	[1]
BL21(DE3)	F^- *omp*T *hsd*S(r_B^- m_B^-)*gal dcm*(DE3)	Takara
***Actinosynnema pretiosum* ssp.**		
ATCC 31280	Wild-type ansamitocin P-3 producing strain	ATCC
WXR-24	ATCC 31280Δ*ansa30*Δ*mec-pks7*::*kas*Op^*-*asm10*	[2]
HQG-1	ATCC 31280::pLQ1550	This study
HQG-3	ATCC 31280::pLQ1552	This study
HQG-6	ATCC 31280::pLQ1555	This study
HQG-7	ATCC 31280::pLQ1556	This study
HQG-9	ATCC 31280::pLQ1558	This study
HQG-12	ATCC 31280::pLQ1561	This study
HQG-13	ATCC 31280::pLQ1562	This study
HQG-14	ATCC 31280::pLQ1563	This study
HQG-16	ATCC 31280::pLQ1565	This study
HQG-17	ATCC 31280::pLQ1566	This study
HQG-19	ATCC 31280::pLQ1568	This study
HQG-20	ATCC 31280::pLQ1569	This study
HQG-21	ATCC 31280::pLQ1570	This study
HQG-22	ATCC 31280::pLQ1571	This study
HQG-23	WXR-24::pLQ1570	This study
HQG-24	WXR-24::pLQ1562	This study
HQG-25	WXR-24::pLQ1556	This study

Supplementary Table 2. Plasmids used in this study and their properties and sources

Plasmids	Features	Sources
pSET152	ØC31 *int, attP, oriT, aac(3)IV*	[1]
pLQ648	pSET152-derived plasmid for the expression of *APASM_1068* under the control of *kas*Op*	[2]
pJTU1278	*rep*-pIJ*101*, *tsr*, *oriT*	[3]
pET30a	pBR322 replicon, P_{T7}, His_6-Tag, Km^R	[4]
pLQ1550	pJTU1278-derived plasmid for *asm7* deletion	This study
pLQ1552	pJTU1278-derived plasmid for *asmA* deletion	This study
pLQ1555	pSET152-derived plasmid for the overexpression of *APASM_1039* under the control of *kas*Op*	This study
pLQ1556	pSET152-derived plasmid for the overexpression of *APASM_1052* under the control of *kas*Op*	This study
pLQ1558	pSET152-derived plasmid for the overexpression of *APASM_1088* under the control of *kas*Op*	This study
pLQ1561	pSET152-derived plasmid for the overexpression of *APASM_2328* under the control of *kas*Op*	This study
pLQ1562	pSET152-derived plasmid for the overexpression of *APASM_5765* under the control of *kas*Op*	This study
pLQ1563	pSET152-derived plasmid for the overexpression of *APASM_5803* under the control of *kas*Op*	This study
pLQ1565	pSET152-derived plasmid for the overexpression of *APASM_6159* under the control of *kas*Op*	This study
pLQ1566	pSET152-derived plasmid for the overexpression of *APASM_6307* under the control of *kas*Op*	This study

pLQ1568	pSET152-derived plasmid for the overexpression of *APASM_6428* under the control of *kasO*p*	This study
pLQ1569	pSET152-derived plasmid for the overexpression of *APASM_6814/6815* under the control of *kasO*p*	This study
pLQ1570	pSET152-derived plasmid for the overexpression of *APASM_6915* under the control of *kasO*p*	This study
pLQ1571	pSET152-derived plasmid for the overexpression of *APASM_6958* under the control of *kasO*p*	This study
pLQ1572	pET30a-derived plasmid for the overexpression of Asm7 in BL21(DE3)	This study
pLQ1573	pET30a-derived plasmid for the overexpression of dTGD in BL21(DE3)	This study
pLQ1574	pET30a-derived plasmid for the overexpression of FDTS in BL21(DE3)	This study
pLQ1575	pET30a-derived plasmid for the overexpression of ALDH in BL21(DE3)	This study

Supplementary Table 3. Primers used in strain constructions

Primers [a]	Sequences (5′–3′) [b]
deasm7-L-F	AAA GGATCC AGAGCCAGGTCTCCAGGTCGG, *Bam*HI
deasm7-L-R	AAA GAATTC GGTGCGCTCGTCGTCGATCAA, *Eco*RI
deasm7-R-F	AAA GAATTC GCGGAGTTCGCCGAGCTGTTC, *Eco*RI
deasm7-R-R	AAA AAGCTT TCGGTCGTGCTGCCGTTCTGC, *Hin*dIII
deasm7-YZ-L	ATGTCAACAGCCCTGTCAGTC
deasm7-YZ-R	TGCTCTCGCTCGACGTCGCTC
deAsmA-L-F	AA GGATCC CTCGCTCAAGTCCAACATCG, *Bam*HI
deAsmA-L-R	AA GAATTC GTAGCGCCAGGAGTCGAG, *Eco*RI
deAsmA-R-F	AA GAATTC GAGACCACCACCGTCGTCA, *Eco*RI
deAsmA-R-R	AA AAGCTT GTCGGACAGCCTCTCCAG, *Hin*dIII
deAsmA-YZ-L	GTGTTCGTGGAGGTCGGG
deAsmA-YZ-R	GTGGGGTCCTGGATGTCC
SAHgbd-F	GCG CATATG ATGACCTTGCGCCTGCAC, *Nde*I
SAHgbd-R	CCG GAATTC CTACCGCAGCCGGGCCGCGAT, *Eco*RI
GBD-kasopF	GTGCGGTGTTGTAAAGTCGT
PTRgbd-F	GCG CATATG ATGATCGAACGAGCCCGG, *Nde*I
PTRgbd-R	CCG GAATTC TCAGCCGTCGAGCCTGCC, *Eco*RI
MAAgbd-F	GCG CATATG GTGGCGGCACCCAACCTC, *Nde*I
MAAgbd-R	CCG GAATTC TCAGGGGTGTTACGACCT, *Eco*RI
FDTSgbd-F	GCG CATATG GTGCAGTTGATCGCGAAG, *Nde*I
FDTSgbd-R	CCG GAATTC TCACCCCTCCGCCACCAG, *Eco*RI
PurSgbd-F	GCG CATATG ATGAGGGTCGGCGTCATC, *Nde*I
PurSgbd-R	CCG GAATTC TCAGTCGAACAGCGCCGGGA, *Eco*RI
dTGDgbd-F	GCG CATATG ATGCGGGTGCTGGTTACA, *Nde*I
dTGDgbd-R	CCG GAATTC TCAGCGGGCCAGGGAGGC, *Eco*RI
ALDHgbd-F	GCG CATATG ATGGCGAAGTACGCGGCA, *Nde*I
ALDHgbd-R	CCG GAATTC TCAGAAGAAGCCCTGCGC, *Eco*RI
IIRgbd-F	GCG CATATG ATGAGCCGACCCGTCACG, *Nde*I
IIRgbd-R	CCG GAATTC TCACTCCTTGACGGAGAA, *Eco*RI
PhnBgbd-F	GCG CATATG ATGCGCTTCATGGTCCTG, *Nde*I
PhnBgbd-R	CCG GAATTC TCAGCCCCGGTTCTCGGT, *Eco*RI
ISOgbd-F	GCG CATATG ATGGGCAAGATCAAGGTT, *Nde*I
ISOgbd-R	CCG GAATTC TCAGCCCGCCATCTTCTT, *Eco*RI
Glxgbd-F	GCG CATATG GTGCCGGGCATGGGGCTG, *Nde*I
Glxgbd-R	CCG GAATTC TCACCCCGCCTTCGACAC, *Eco*RI

Primers [a]	Sequences (5′ – 3′) [b]
MerRgbd-F	GCG CATATG GTGCGGTACTACTCGATC, *Nde*I
MerRgbd-R	CCG GAATTC TCAGCCGATCGACGCCAG, *Eco*RI
Asm7-F	AA GAATTC GTGACGTTGATCGACGACGA, *Eco*RI
Asm7-R	AA AAGCTT TCACCCGTCCGTGCGCGGCT, *Hin*dIII
dTGD-F	AA GAATTC ATGCGGGTGCTGGTTACA, *Eco*RI
dTGD -R	AA AAGCTT TCAGCGGGCCAGGGAGGC, *Hin*dIII
FDTS-F	AA GAATTC GTGCAGTTGATCGCGAAG, *Eco*RI
FDTS-R	AA AAGCTT TCACCCCTCCGCCACCAG, *Hin*dIII
ALDH-L	AA GAATTC ATGGCGAAGTACGCGGCA, *Eco*RI
ALDH-R	AA AAGCTT TCAGAAGAAGCCCTGCGC, *Hin*dIII

[a] F indicates forward primer, and R indicates reverse primer.

[b] The underlined nucleotides indicate restriction sites used for cloning with pSET152, pLQ648 or pET30a.

Supplementary Table 4. Medium components

Media	Components (*w/v*) [a]
Luria-Bertani broth	0.5% yeast extract, 1% tryptone, 1% NaCl
YMG medium	0.4% yeast extract, 1% malt extract, 0.4% glucose and 1.5% agar
S1 medium	0.5% yeast extract, 3% tryptone soya broth and 10.3% sucrose
S2 medium	0.8% yeast extract, 3% tryptone soya broth, 10.3% sucrose, isobutanol 500 μL/L and isopropanol 500 μL/L, pH 7.5
YMS medium	1.6% yeast extract, 1% malt extract, 10.3% sucrose, 2.5% starch, isopropanol 12 mL/L, isobutanol 5 mL/L and $MgCl_2$ 2 mM, pH 7.5
YMV medium	2.4% yeast extract, 1% malt extract, 10.3% sucrose, isopropanol 12 mL/L, isobutanol 5 mL/L, $MgCl_2$ 2 mM, L-valine 40 mM, pH 7.5

[a] Yeast extract, malt extract, tryptone soya broth and tryptone were purchased from Oxoid. NaCl, glucose, sucrose, agar, isobutanol, isopropanol, starch and $MgCl_2$ were purchased from Sinopharm Chemical Reagent Co.

Supplementary Table 5. Target proteins identified from the chemoproteomic assay

	Protein-coding genes	Annotation
1	*APASM_1039*	Prenyltransferase (PTR)
2	*APASM_1052*	Aldehyde dehydrogenase (ALDH)
3	*APASM_1088*	Membrane alanine aminopeptidase (MAA)
4	*APASM_2328*	Inosose isomerase (IIR)
5	*APASM_3207*	*O*-Methyltransferase (ASM7)
6	*APASM_5765*	Flavin-dependent thymidylate synthase (FDTS)
7	*APASM_5803*	Glyoxalase family protein (GLX)
8	*APASM_6159*	Regulator (PhnB)
9	*APASM_6307*	dTDP-glucose 4,6-dehydratase (dTGD)
10	*APASM_6428*	Isocitrate dehydrogenase (ISO)
11	*APASM_6814*/*APASM_6815*	Phosphoribosylformylglycinamidine synthase (PurS)
12	*APASM_6915*	Transcriptional regulator (MerR)
13	*APASM_6958*	*S*-adenosylhomocysteine deaminase (SAH)

Supplementary Table 6. Binding constants of target proteins with ansamitocin P-3

Immobilized proteins	ka (1/Ms)	kd (1/s)	K_D (M)
Asm7	8.33×10^{3}	2.99	3.59×10^{-4}
dTGD	2.24×10^{3}	8.06×10^{-1}	3.60×10^{-4}
FDTS	1.47×10^{4}	9.78×10^{-1}	6.65×10^{-5}
ALDH	3.48×10^{4}	6.86×10^{-1}	1.97×10^{-5}

Affinity constant (K_D)= dissociation constant (kd) / association constant (ka).

Supplementary Table 7. 95% confidence interval of k_{cat}, K_m and K_I of dTGD

	95% CI of k_{cat} (1/min)	95% CI of K_m (mM/min)	95% CI of K_I (mM)
DMSO	39.25 to 48.18	0.12 to 0.20	0.92 to 1.34
0.31 mM AP-3	30.80 to 39.05	0.12 to 0.23	-
0.63 mM AP-3	25.26 to 35.02	0.12 to 0.28	-

Supplementary Table 8. 95% confidence interval of k_{cat}, K_m and K_I of FDTS

	95% CI of k_{cat} (1/min)	95% CI of K_m (mM/min)	95% CI of K_I (mM)
DMSO	53.99 to 61.51	0.53 to 0.82	0.047 to 0.061
0.02 mM AP-3	41.00 to 44.85	0.55 to 0.74	-
0.04 mM AP-3	29.23 to 37.34	0.46 to 1.02	-

Supplementary Table 9. 95% confidence interval of k_{cat}, K_m and K_I of ALDH

	95% CI of k_{cat} (1/min)	95% CI of K_m (mM/min)	95% CI of K_I (mM)
DMSO	7.36 to 9.46	0.071 to 0.150	0.66 to 1.06
0.31 mM AP-3	4.96 to 6.034	0.048 to 0.092	-
0.63 mM AP-3	3.64 to 4.38	0.037 to 0.070	-

SUPPLEMENTARY FIGURES

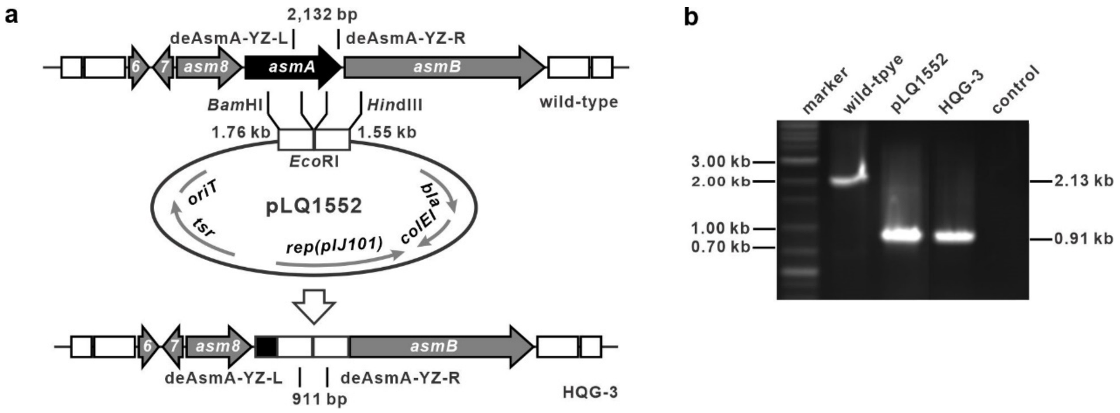

Supplementary Figure 1. Construction and verification of the *asmA* deletion mutant HQG-3.

a, Diagram of homologous double crossovers for *asmA* deletion in ATCC 31280. **b**, Verification of the *asmA* deletion mutant HQG-3 by gel electrophoresis of PCR products; wild-type, ATCC 31280; HQG-3, *asmA* deletion mutant; pLQ1552, plasmid used to generate *asmA* deletion; control, ddH_2O.

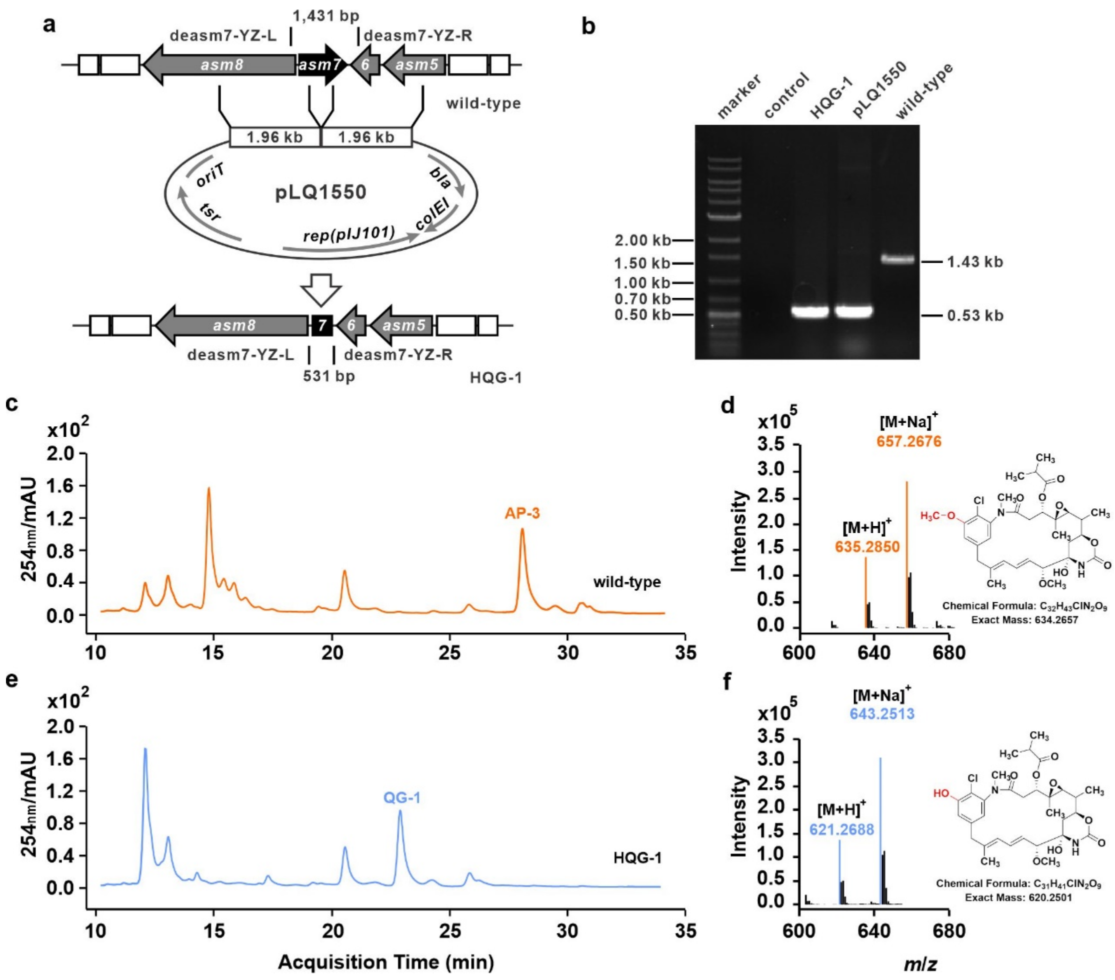

Supplementary Figure 2. Construction and verification of *asm7* deletion mutant HQG-1.

a, Diagram of homologous double crossovers for *asm7* deletion in ATCC 31280. **b**, Verification of the *asm7* deletion mutant HQG-1 by gel electrophoresis of PCR products; wild-type, ATCC 31280; HQG-1, *asm7* deletion mutant; pLQ1550, plasmid used to generate *asm7* deletion; control, ddH_2O. **c**, HPLC profile for the fermentation products of ATCC 31280. **d**, MS confirmation of compounds AP-3, *m/z* = 635.2850 $[M+H]^+$ for AP-3. **e**, HPLC profile for the fermentation products of the mutant strain HQG-1. **f**, MS confirmation of compounds QG-1, *m/z* = 621.2688 $[M+H]^+$ for QG-1.

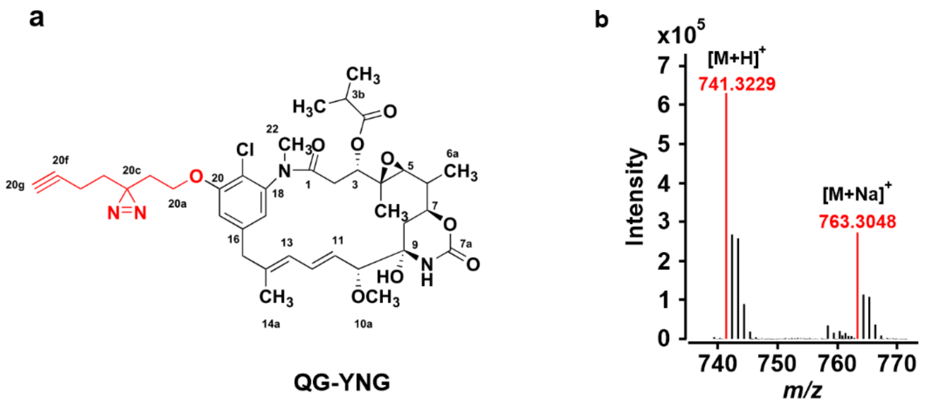

Supplementary Figure 3. Mass spectrometry analysis of QG-YNE.

a, The structure of compound QG-YNE. **b,** ESI-MS result for QG-YNE. *m/z* = 741.3229 $[M+H]^{+}$, *m/z*= 763.3048 $[M+Na]^{+}$.

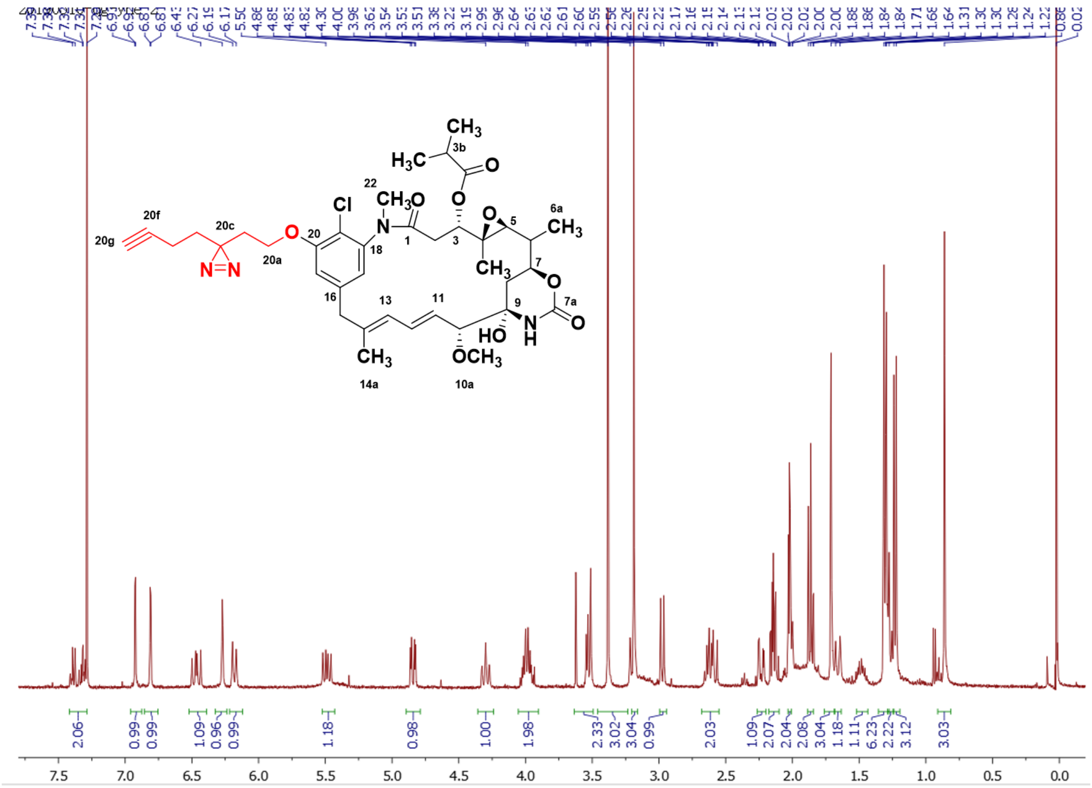

Supplementary Figure 4. ¹H NMR spectrum for QG-YNE.

¹H NMR (400 MHz, $CDCl_3$) δ 7.42 – 7.29 (m, 2H, 9-OH, 9-NH), 6.92 (d, *J* = 1.6 Hz, 1H, 17-CH), 6.81 (d, *J* = 1.8 Hz, 1H, 21-CH), 6.47 (dd, *J* = 15.3, 10.9 Hz, 1H, 11-CH), 6.27 (s, 1H, 13-CH), 6.18 (d, *J* = 10.8 Hz, 1H, 12-CH), 5.49 (dd, *J* = 15.4, 9.0 Hz, 1H, 3-CH), 4.84 (dd, *J* = 12.0, 3.2 Hz, 1H, 10-CH), 4.30 (t, *J* = 10.5 Hz, 1H, 7-CH), 4.05 – 3.90 (m, 2H, 20a-CH_2), 3.55 (dd, *J* = 24.7, 19.8 Hz, 2H, 15-CH_2), 3.38 (s, 3H, 22-N-CH_3), 3.19 (s, 3H, 10a-CH_3), 2.98 (d, *J* = 9.7 Hz, 1H, 20g-CH), 2.61 (m, 2H, 2-CH_2), 2.23 (dd, *J* = 13.9, 3.0 Hz, 1H, 3b-CH), 2.14 (m, 2H, 20e-CH_2), 2.03 – 2.01 (m, 2H, 20b-CH_2), 1.86 (m, 2H, 20d-CH_2), 1.71 (s, 3H, 14a-CH_3), 1.66 (d, *J* = 13.3 Hz, 1H, 5-CH), 1.52 – 1.44 (m, 1H, 6-CH), 1.30 (dd, *J* = 8.7, 6.5 Hz, 6H, 3c-CH_3, 3d-CH_3), 1.28 (m, 2H, 8-CH_2), 1.23 (d, *J* = 6.7 Hz, 3H, 4a-CH_3), 0.86 (s, 3H, 6a-CH_3).

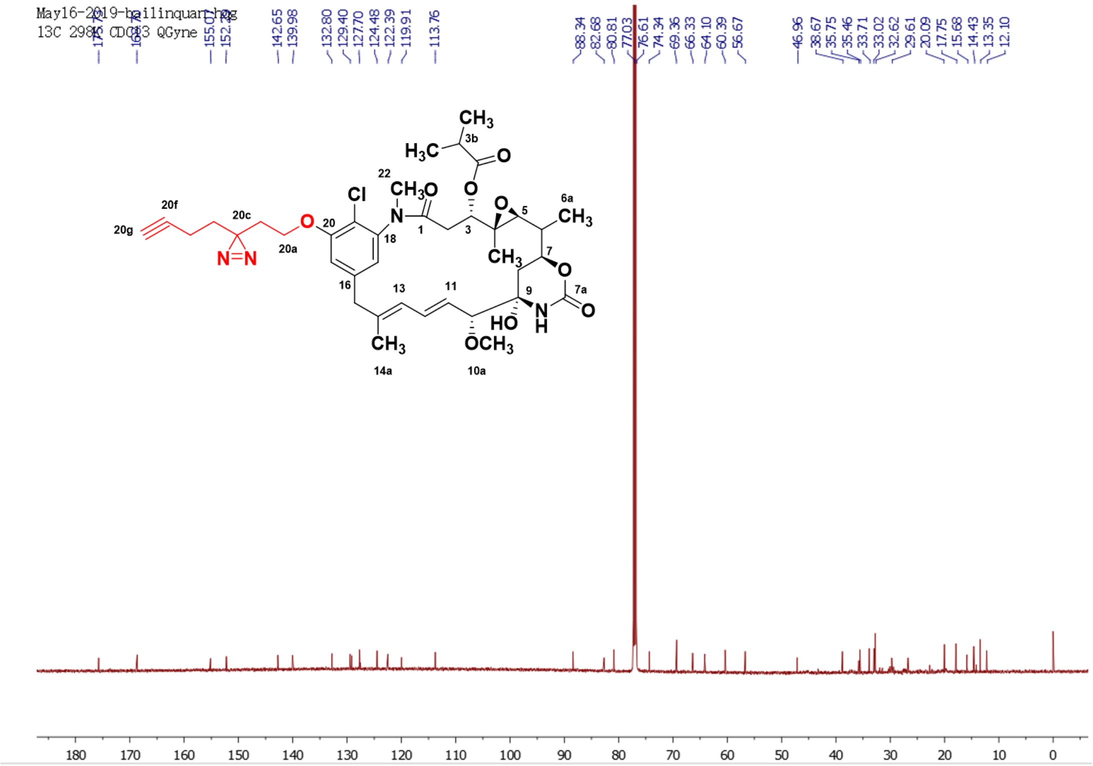

Supplementary Figure 5. ^{13}C NMR spectrum for QG-YNE.

^{13}C NMR (151 MHz, $CDCl_3$) δ 175.73, C-3a, 168.70, C-1,155.07, C-7a, 152.29, C-20, 142.65, C-16, 139.98, C-18, 132.80, C-11, 119.91, C-19, 129.07, C-14, 127.70, C-12, 124.33, C-13, 122.35, C-17, 113.76, C-21, 82.68, C-9, 80.81, C-20f, 69.32, C-20c, 60.39, C-4, 64.10, C-20a, 46.96, C-15, 35.80, C-20b, 33.02, C-20e, 32.62, C-8, 29.61, C-2, 13.35, C-20d, 88.40, C-7, 77.23, C-5, 76.73, C-3, 74.34, C-10, 66.33, C-20g, 56.67, C-10a, 38.67, C-6, 35.46, C-22, 33.71, C-3b, 20.09, C-3d, 17.75, C-3c, 15.68, C-14a, 14.43, C-6a, 12.10, C-4a.

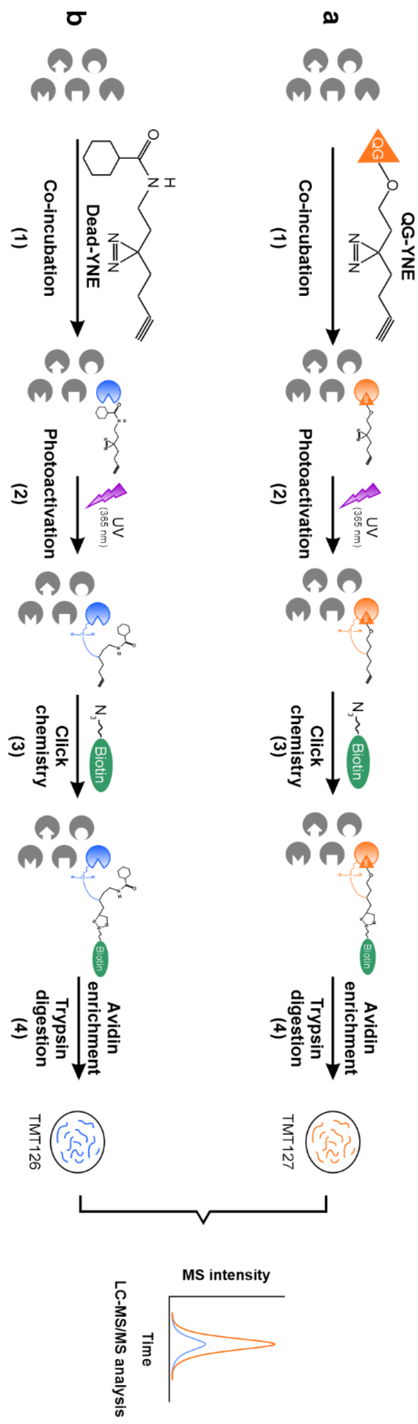

Supplementary Figure 6. Design of the photoaffinity binding protein profiling experiments to identify the ansamitocin P-3 binding protein spectrum in ATCC 31280 by proteomic analysis.

a, b, Schematic diagrams representing the (**a**) experimental group containing QG-YNE and (**b**) control group containing the dead-YNE. **Step 1**: Co-incubation of probe and whole cellular proteins. **Step 2**: photoactivated covalent connection between ansamitocin and binding proteins. **Step 3**: Protein-small molecule complexes are tagged to biotin by click

reaction and captured by streptavidin. **Step 4**: Tryptic digestion of the beads-bound proteins and labeling with TMT-126/127.

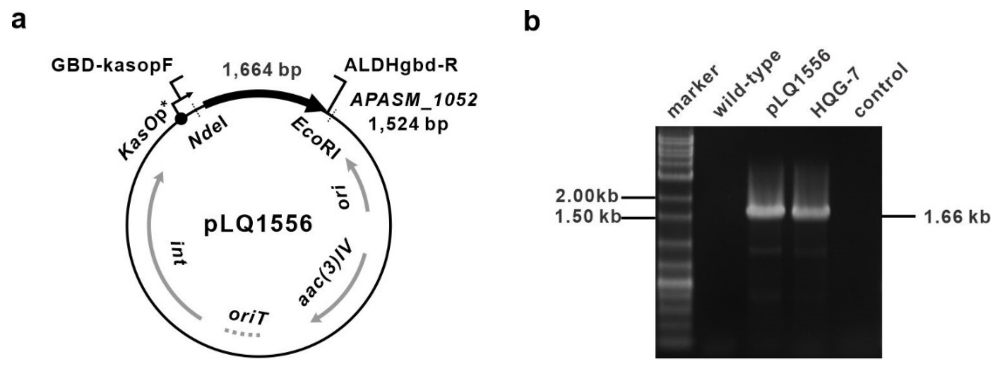

Supplementary Figure 7. Construction and verification of the *APASM_1052* overexpression strain.

a, Schematic diagram for the construction of the *APASM_1052* overexpression plasmid pLQ1556 containing the *kas*Op* promoter. **b,** Verification of the *APASM_1052* overexpression strain HQG-7 by gel electrophoresis of PCR products; wild-type, ATCC 31280; HQG-7, *APASM_1052* overexpression strain HQG-7; pLQ1556, plasmid used to generate *APASM_1052* overexpression; control, ddH_2O. All overexpression strains described in this study were similarly constructed.

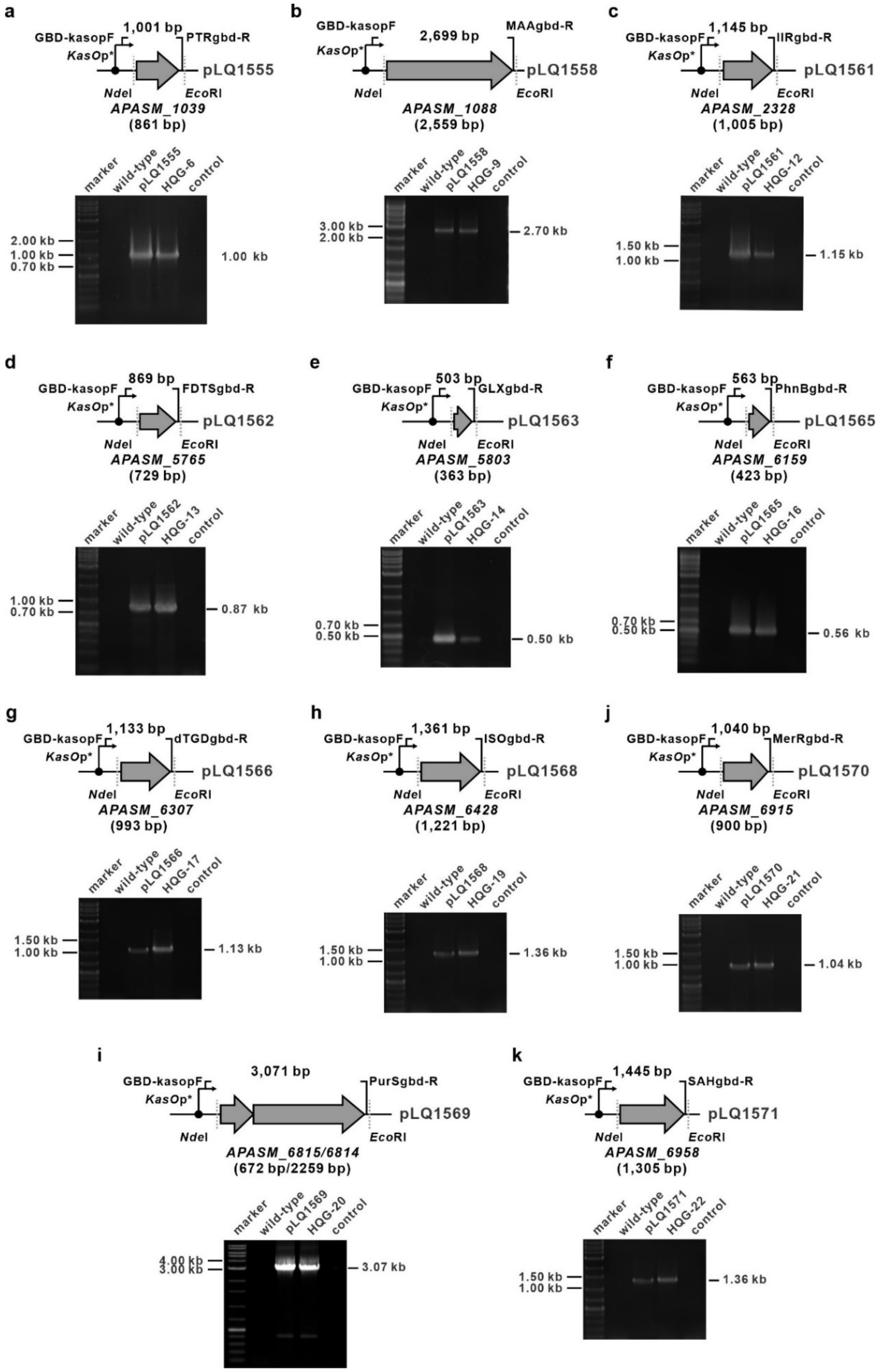

Supplementary Figure 8. Verification of overexpression strains by gel electrophoresis of PCR products.

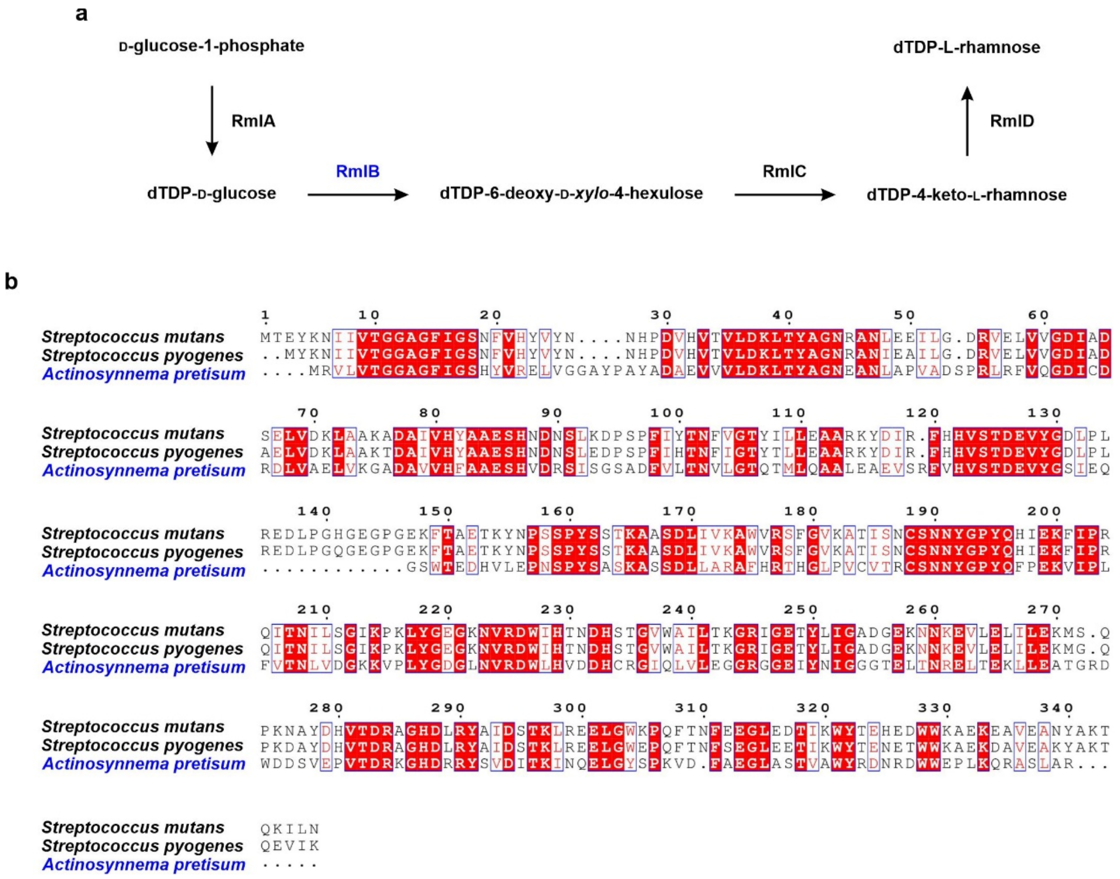

Supplementary Figure 9. Amino acid sequence alignment revealed that dTGD in ATCC 31280 participates in dTDP-L-rhamnose biosynthesis.

a, dTDP-L-rhamnose biosynthetic pathway in bacteria. dTGD is a homologous protein of RmlB. **b**, Protein sequence alignment and identity matrix of dTGD homologs. As reported, RmlB proteins in *Streptococcus mutans* and *Streptococcus pyogenes* participate in dTDP-L-rhamnose biosynthesis, and their sequence identities with dTGD of ATCC 31280 are 40.61% and 40.91%, respectively. Identical residues are colored in white against a red background, and those residues with similar properties are colored in red within blue frames.

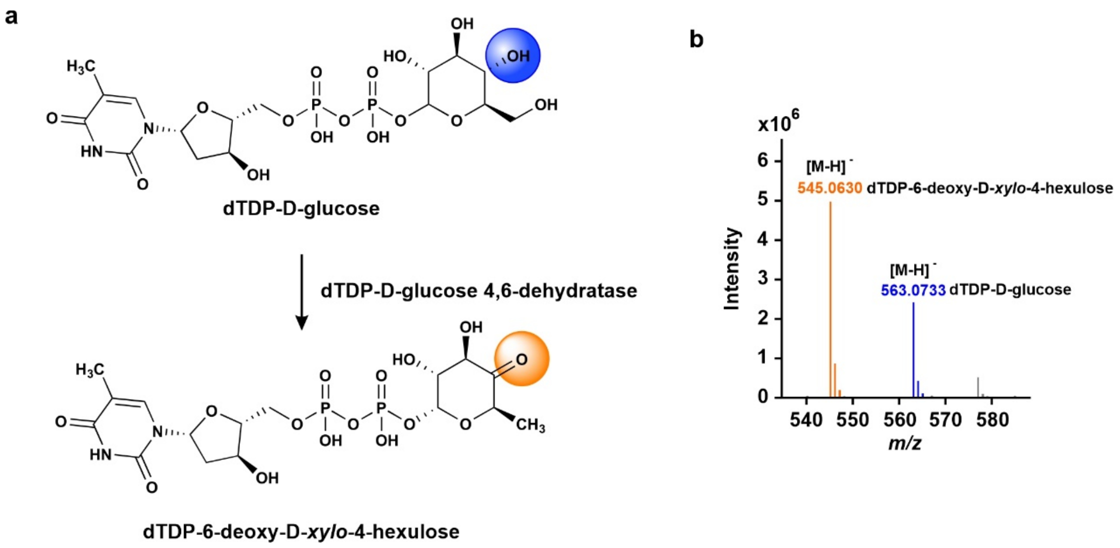

Supplementary Figure 10. In vitro reaction for dTGD with dTDP-D-glucose and NAD+ as substrates.

a, dTGD catalyzes the formation of dTDP-6-deoxy-D-*xylo*-4-hexulose from dTDP-D-glucose. **b,** MS detection of product formation catalyzed by dTGD. *m/z* = 545.0630 $[M-H]^-$ for dTDP-6-deoxy-D-*xylo*-4-hexulose, *m/z*= 563.0733 $[M-H]^-$ for dTDP-D-glucose. **c**, Standard curve for dTDP-6-deoxy-D-*xylo*-4-hexulose at A_{320}. Calibration equation: y = 0.9232 x, R^2 = 0.9974.

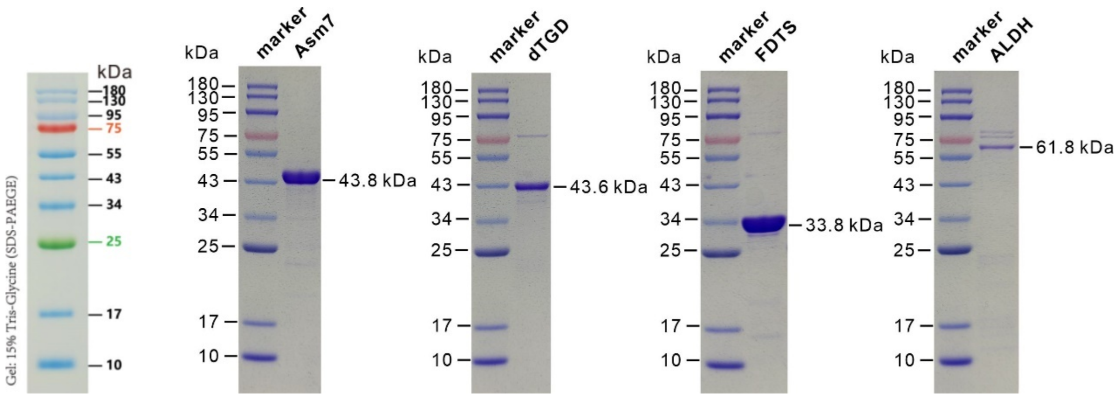

Supplementary Figure 11. Overexpression of recombinant His-tagged target proteins in *E. coli* BL21(DE3) and analysis by SDS-PAGE.

10	20	30	40	50	60
MRVLVTGGAG	FIGSHYVREL	VGGAYPAYAD	AEVVVLDKLT	YAGNEANLAP	VADSPRLRFV
70	80	90	100	110	120
QGDICDRDLV	AELVKGADAV	VHFAAESHVD	RSISGSADFV	LTNVLGTQTM	LQAALEAEVS
130	140	150	160	170	180
RFVHVSTDEV	YGSIEQGSWT	EDHVLEPNSP	YSASKASSDL	LARAFHRTHG	LPVCVTRCSN
190	200	210	220	230	240
NYGPYQFPEK	VIPLFVTNLV	DGKKVPLYGD	GLNVRDWLHV	DDHCRGIQLV	LEGGRGGEIY
250	260	270	280	290	300
NIGGGTELTN	RELTEKLLEA	TGRDWDDSVE	PVTDRKGHDR	RYSVDITKIN	QELGYSPKVD
310	320	330			
FAEGLASTVA	WYRDNRDWWE	PLKQRASLAR			

Supplementary Figure 12. QG-YNE specific binding at 314-DNRDWWEPLKQR-325 of dTGD.

Red font, QG-YNE-modified sites; highlighted in yellow, QG-YNE-modified peptide.

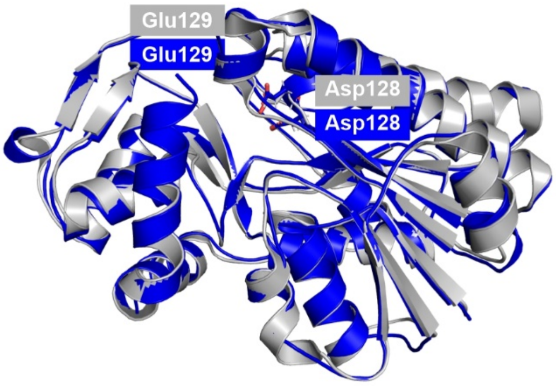

Supplementary Figure 13. Structure alignment of homologous proteins for dTGD and published crystal structure.

Blue, architectures simulated with the AlphaFold2 server. Grey, protein structures obtained from the PDB database. Strictly conserved amino acids in active pocket are marked in abbreviated form.

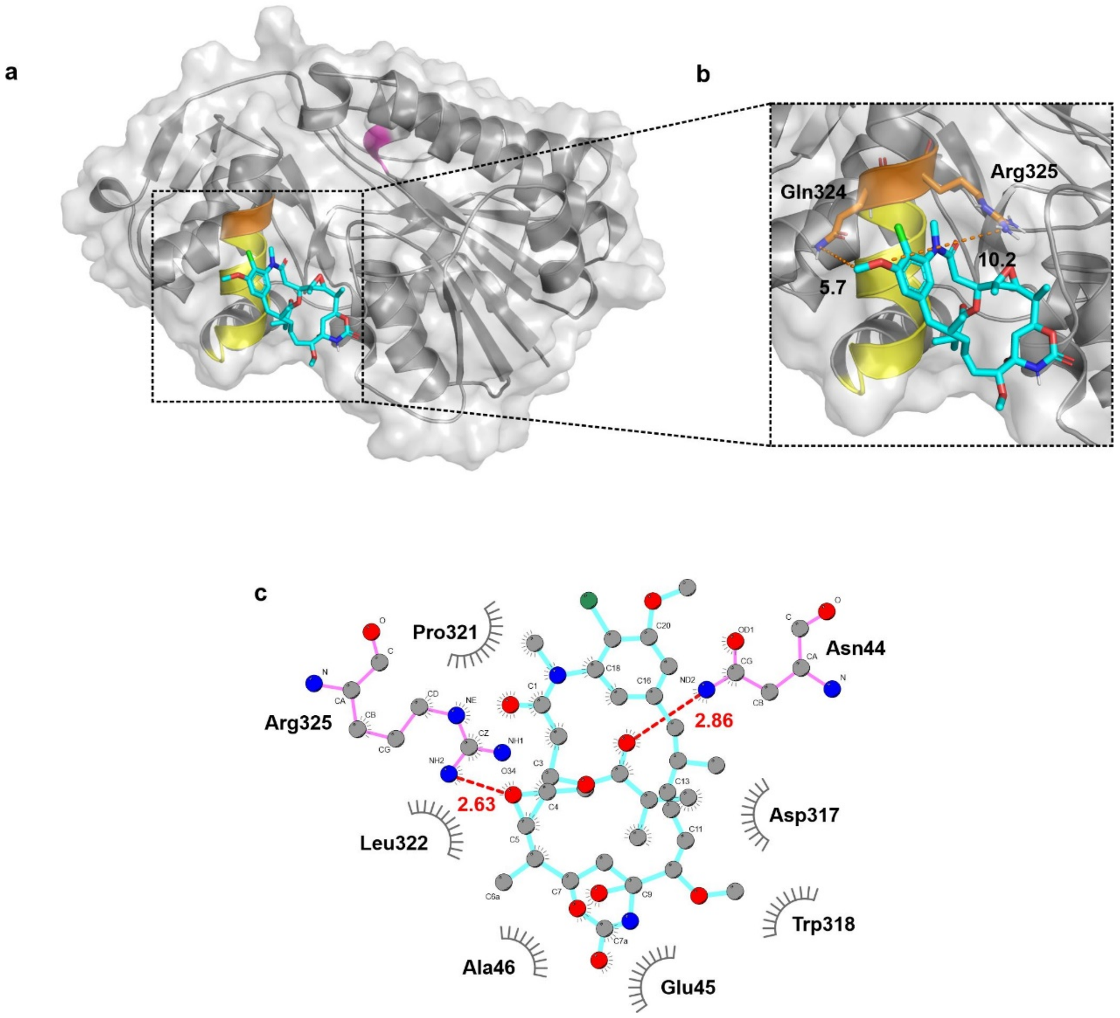

Supplementary Figure 14. Interaction of dTGD and AP-3 revealed by docking analysis.

a, Overall view of the AP-3 and dTGD homo-monomer complex. Light grey, dTGD homo-monomer interacting with AP-3; sky blue, AP-3; yellow, peptide labeled by QG-YNE; orange, amino acid modified by QG-YNE; violet, conserved amino acid of dTGD. **b**, Close-up view of the interaction observed between AP-3 and the dTGD homo-monomer. The key amino acid residues of dTGD are shown by stick representation and are labeled. The distances (Å) between the modified amino acids and AP-3 are indicated by dashed orange lines. The oxygen at C20 of AP-3 is near the Gln324 and Arg325 residues with distances of 5.70 Å and 10.20 Å. **c**, 2D display model of key amino acid residues of dTGD interacting with AP-3. The amino residues Asn44 and Arg325 provide the hydrogen-bonding interactions with the C4-C5 epoxy group and C3 ketonic oxygen of AP-3 with distances of 2.86 Å and 2.63 Å. The macrolactam ring of AP-3 is

inserted into the hydrophobic pocket of dTGD, and key amino acid residues of dTGD interacting with AP-3 are identified as Glu45, Ala46, Asp317, Trp318, Pro321 and Leu322. Red dashed lines, hydrogen bonds between key amino acid residues of dTGD and AP-3. The bond lengths (Å) are indicated by the numbers beside the dotted lines. Amino acids of dTGD that interact hydrophobically with AP-3 are marked in abbreviated form.

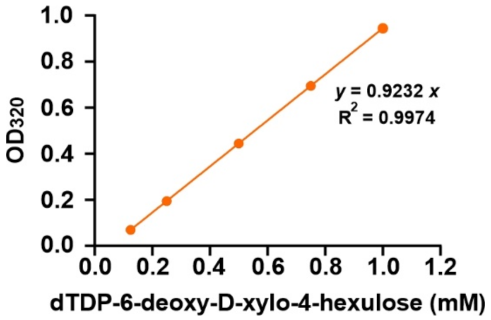

Supplementary Figure 15. Standard curve for dTDP-6-deoxy-D-*xylo*-4-hexulose at A_{320}. Calibration equation: $y = 0.9232\,x$, $R^2 = 0.9974$.

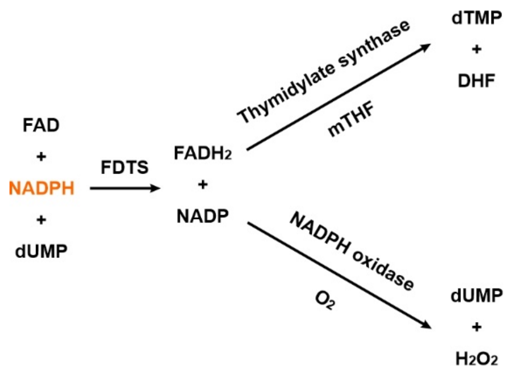

Supplementary Figure 16. Thymidylate synthase and NADPH oxidase activities of FDTS.

FDTS initially catalyzes the formation of the $FADH_2$ cofactor from FAD, with the consumption of two-equivalent NADPH and the presence of dUMP, and then transfers a methylene group from mTHF to dUMP to generate dTMP and 7,8-dihydrofolate (DHF). When the methylene group donor mTHF is absent from the reactions, FDTS only functions as an NADPH oxidase to generate $FADH_2$ and two-equivalent NADP, followed by an immediate conversion of $FADH_2$ into FAD and H_2O_2 in the presence of O_2. **b**, Standard curve for NADPH at A_{340}. Calibration equation: $y = 0.8404\ x + 0.0018$, $R^2 = 0.9999$.

10	20	30	40	50	60
VQLIAKTEFF	PPSDVPWSTD	AEGGEALAEF	AGRACYQSWS	KPNPATATNE	AYLRHIIEVG
70	80	90	100	110	120
HLSVLEHGSV	SFYITGISRS	LTHELIRHRH	FSYSQLSQRY	VPERDAAMVE	PEVIANDPEL
130	140	150	160	170	180
HARFLAAAEA	SVAAYNDLLK	GLEEKFSDVP	SATLRRKQAR	QAARAVLPNA	TETRLVVTGN
190	200	210	220	230	240
YRAWRHFIAM	RATEHADVEI	RALAIECLRH	LQKAAPGAFA	DFAITSLADG	TEVASSPLVA
EG					

Supplementary Figure 17. QG-YNE specific binding at 90-HFSYSQLSQR-99 of FDTS.

Red font, QG-YNE-modified site; highlighted in yellow, QG-YNE-modified peptide.

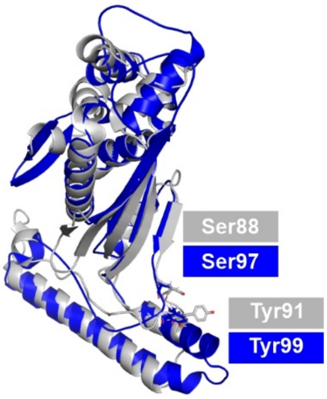

Supplementary Figure 18. Structure alignment of homologous proteins for FDTS and published crystal structure.

Blue, architectures simulated with the AlphaFold2 server. Grey, protein structures obtained from the PDB database. Strictly conserved amino acids in active pocket are marked in abbreviated form.

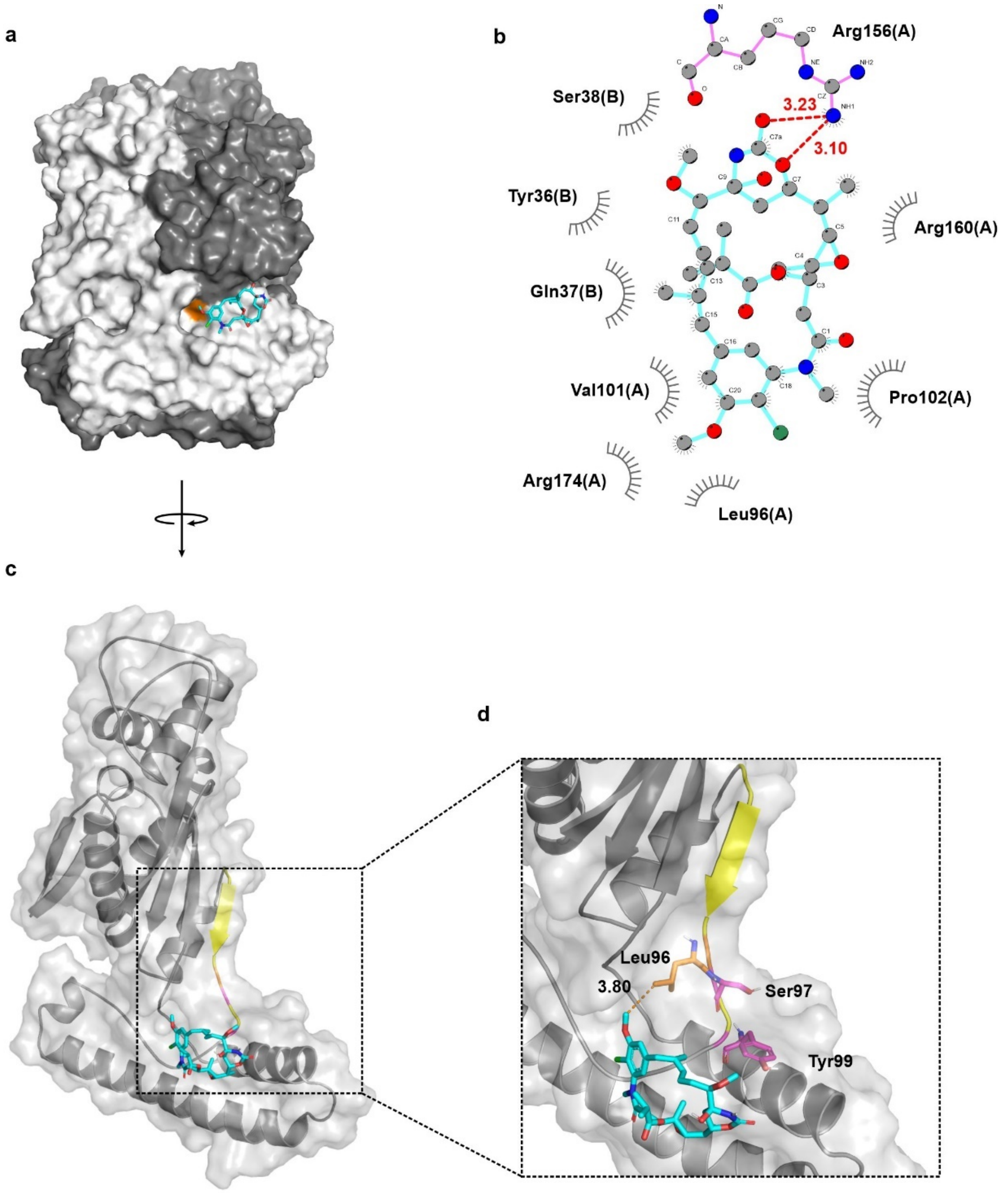

Supplementary Figure 19. Interaction of FDTS and AP-3 revealed by docking analysis.

a, Overall view of the AP-3 and FDTS homo-tetramer complex. Light grey, FDTS homo-monomer interacting with AP-3; dark grey, other monomers of the FDTS tetramer. **b**, 2D display model of key amino acid residues of FDTS interacting with AP-3. The side chain of Arg156 forms hydrogen-bond interactions with the C7a group of AP-3 with distances of 3.23 Å and 3.10 Å. The hydrophobic amino residues towards the hydrophobic region of AP-3 consist of Leu96, Val101, Arg174, Pro102 and Arg160 in the light grey homo-monomer A and Tyr36, Gln37 and Ser38 in the dark grey homo-

monomer B. **c**, Overall view of the AP-3 and FDTS homo-monomer A interactions in different orientations. **d**, Close-up view of the interactions observed between AP-3 and the FDTS homo-monomer. The oxygen at C20 is close to Leu96 by 3.80 Å. Light grey, FDTS homo-monomer interacting with AP-3; sky blue, AP-3; yellow, peptide labeled by QG-YNE; orange, amino acid modified by QG-YNE; violet, conserved amino acid of FDTS.

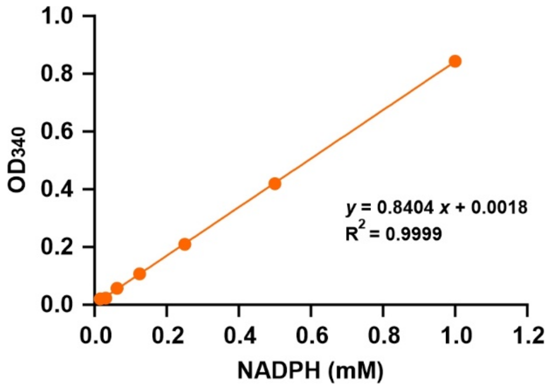

Supplementary Figure 20. Standard curve for NADPH at A_{340}. Calibration equation: $y = 0.8404\,x + 0.0018$, $R^2 = 0.9999$.

a

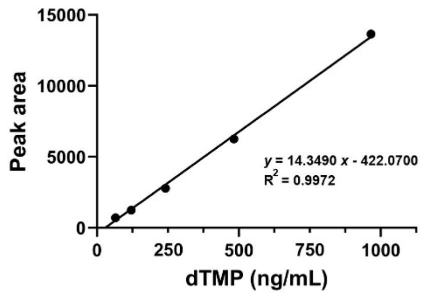

b

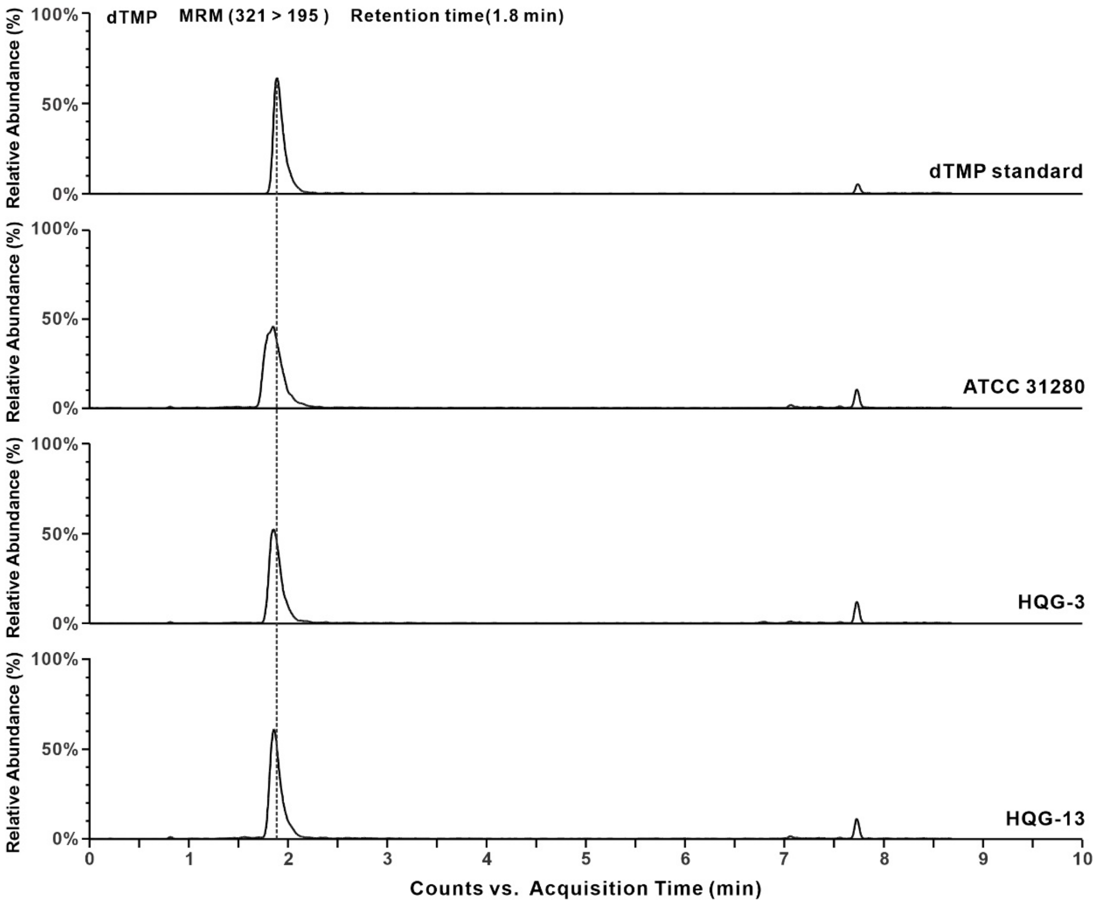

Supplementary Figure 21. Quantitative analysis of dTMP.

a, Standard curve for dTMP. calibration equation: y = 14.349 x – 422.07, R^2 = 0.9972. The dTMP concentrations used to generate the curve were 10, 121, 242, 483 and 966 ng/mL.

b, MRM quantitative signal for compound dTMP in the wild-type ATCC 31280, HQG-3 and HQG-13. Precursor ion and qualitative ion (*m*/*z*) in MRM mode of dTMP were 321>195. The retention time of dTMP was 1.8 min. The average integral signals of dTMP for ATCC 31280, HQG-3 and HQG-13 were 10,641, 11,416 and 12,650, respectively.

10	20	30	40	50	60
MAKYAAPGQP	DSVVSYRSRY	DHFIGGEFTA	PAKGGYFENP	TPVTGETFTE	VARGTAEDVE
70	80	90	100	110	120
RALDAAHGAA	PAWGRTSPAE	RANVLNKIAD	RIEANLEALA	VAETWDNGKA	VRETLAADLP
130	140	150	160	170	180
LAVDHFRYFA	GALRAQEGGI	SQIDENLVAY	HFHEPLGVVG	QIIPWNFPIL	MAVWKLAPAL
190	200	210	220	230	240
AAGNAVVLKP	AEQTPASIMV	LVELIADLLP	PGVLNVVNGF	GVEAGKPLAT	SKRVAKVAFT
250	260	270	280	290	300
GETTTGRLIM	QYASENIIPV	TLELGGKSPN	IFFGDVAAQR	DEFYDKALEG	FTMFALNQGE
310	320	330	340	350	360
VCTCPSRALI	QGSIYEQFLG	DAVERTKAVK	QGHPLDTDTM	IGAQASNDQL	EKILAYIEIG
370	380	390	400	410	420
KAEGATLLTG	GGRADLGGEL	SGGYYVTPTV	FEGDNKMRIF	QEEIFGPVVS	VARFDDYADA
430	440	450	460	470	480
IKIANDTLYG	LGAGVWSRDG	STAYRAGREI	QAGRVWVNNY	HTYPAHAAFG	GYKQSGIGRE
490	500				
NHRMMLDHYQ	QTKNLLVSYA	PGAQGFF			

Supplementary Figure 22. QG-YNE specific binding at 18-SRYDHFIGGEFTAPAK-33 of ALDH.

Red font, QG-YNE-modified site; highlighted in yellow, QG-YNE-modified peptide.

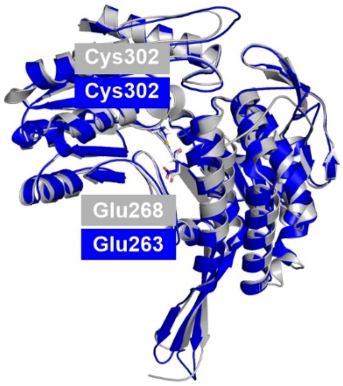

Supplementary Figure 23. Structure alignment of homologous proteins for ALDH and published crystal structure.

Blue, architectures simulated with the AlphaFold2 server. Grey, protein structures obtained from the PDB database. Strictly conserved amino acids in active pocket are marked in abbreviated form.

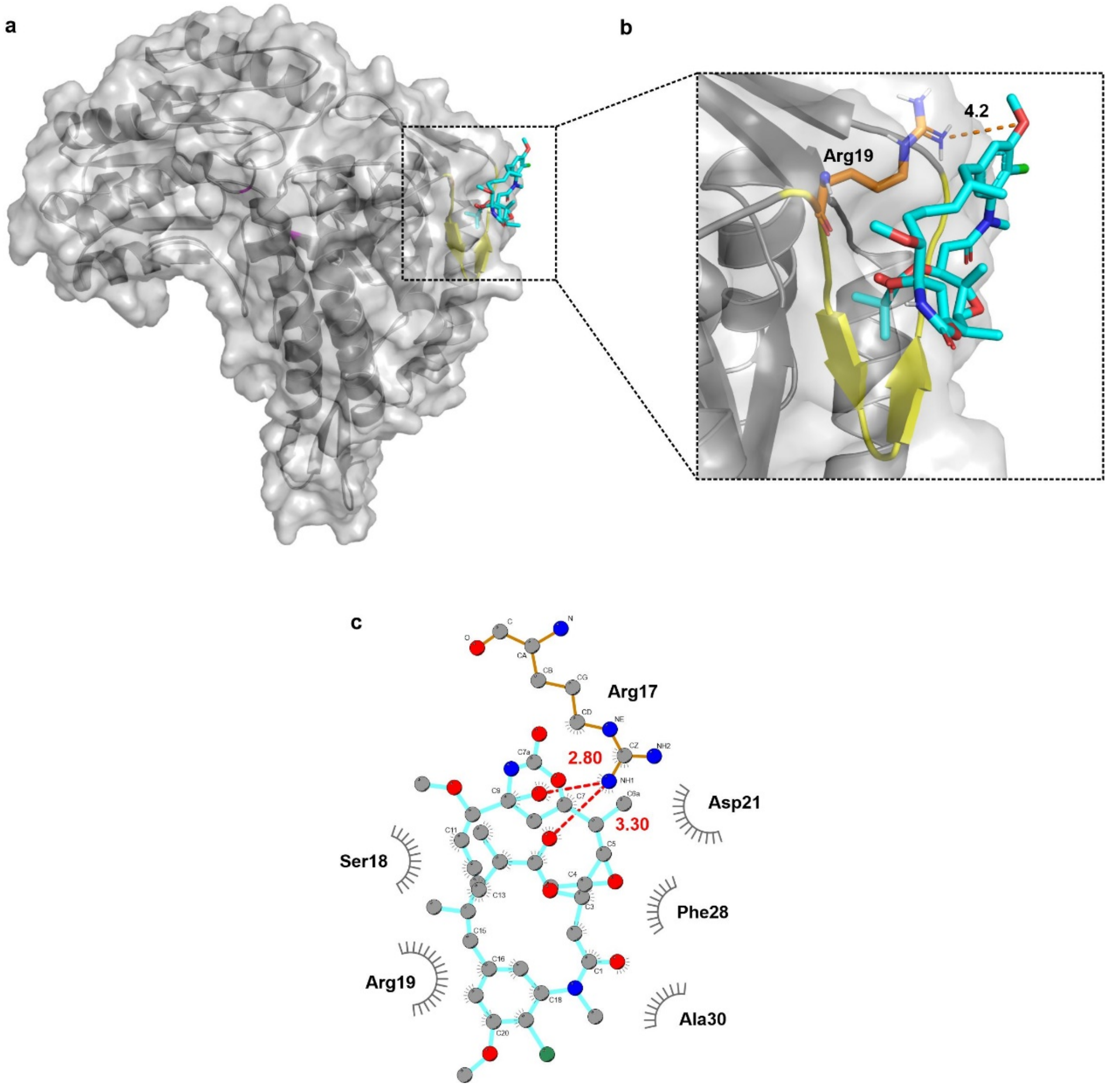

Supplementary Figure 24. Interaction of ALDH and AP-3 revealed by docking analysis.

a, Overall view of the AP-3 and ALDH homo-monomer complex. **b**, Close-up view of the interaction observed between AP-3 and ALDH homo-monomer. The oxygen at C20 is near Arg19 at a distance of 4.20 Å. Light grey, ALDH homo-monomer interacting with AP-3; sky blue, AP-3; yellow, peptide labeled by QG-YNE; orange, amino acid modified by QG-YNE; violet, conserved amino acid of ALDH. **c**, 2D display model of key amino acid residues of ALDH interacting with AP-3. The carbonyl group of the acetyl moiety at C3 and hydroxy at C9 form hydrogen-bond interactions with residue Arg17 with distances of 3.30 Å and 2.80 Å. The macrolactam ring of AP-3 is attached to the pocket of ALDH through hydrophobic interactions with Ser18, Arg19, Asp21, Phe28 and Aln30.

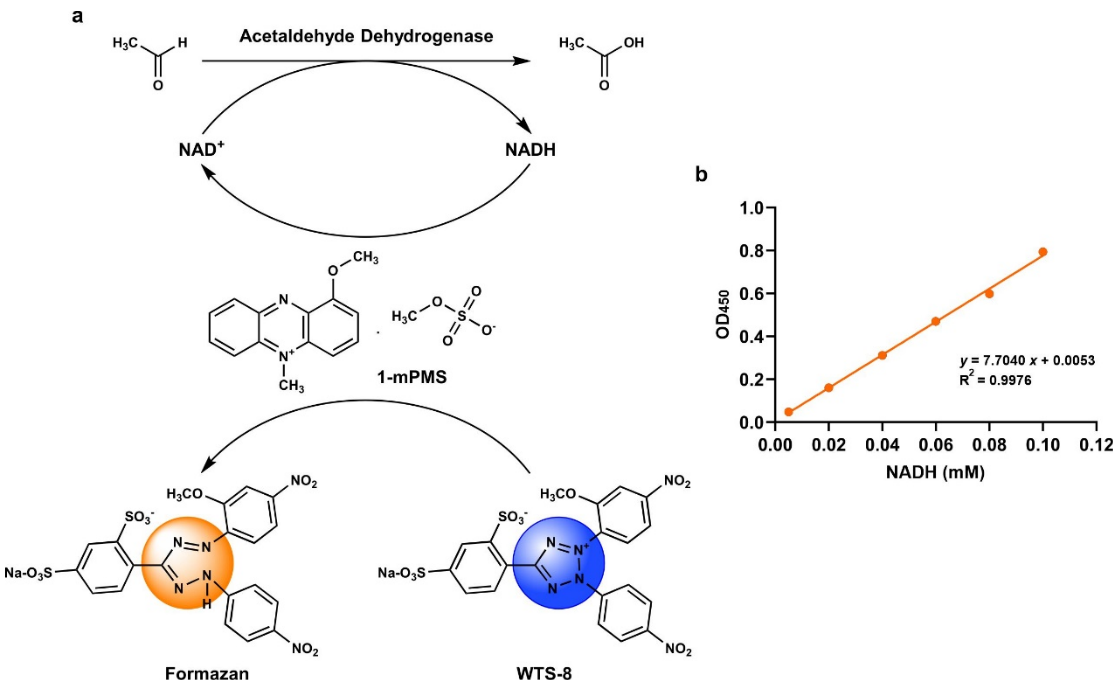

Supplementary Figure 25. Detection of ALDH activity by NADH generation.

a, NAD^+ acts as cofactor for ALDH catalyzed conversion of aldehydes into acids, and the generation of NADH can be used to indicate catalytic activity. In the presence of 1-mPMS, NADH can interact with WTS-8 to generate formazan with a strong absorption at 450 nm. **b**, Standard curve for NADH at A_{450}. Calibration equation: y = 7.7040 x + 0.0053, R^2 = 0.9976.

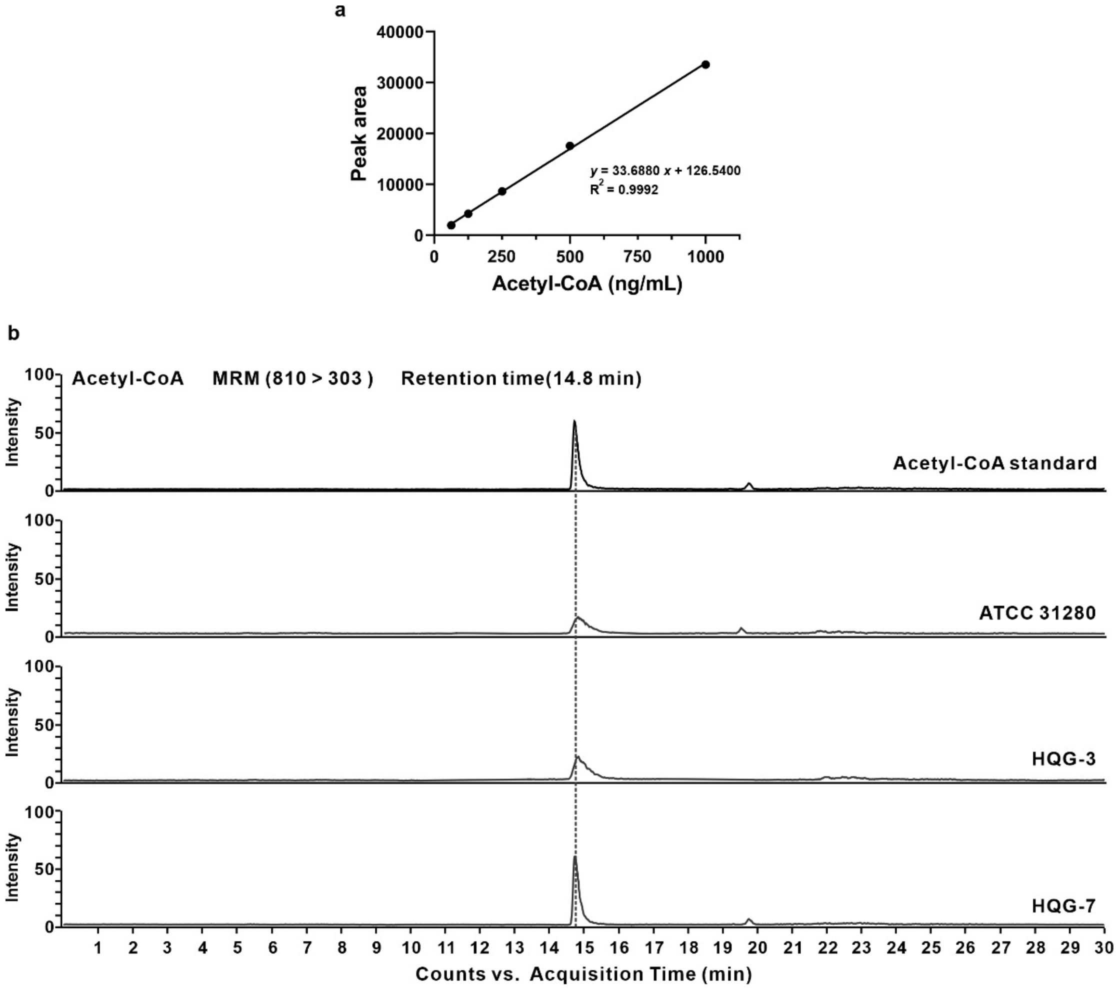

Supplementary Figure 26. Quantitative analysis of acetyl-CoA.

a, Standard curve for acetyl-CoA. Calibration equation: $y = 33.688\ x + 126.54$, $R^2 = 0.9992$. The acetyl-CoA concentrations used to generate the curve were 62.5, 125, 250, 500 and 1,000 ng/mL. **b**, MRM quantitative signal of compound acetyl-CoA in the wild-type ATCC 31280, HQG-3 and HQG-7. Precursor ion and qualitative ion (*m*/*z*) in MRM mode of acetyl-CoA were 810>303. The retention time of acetyl-CoA was 15 min. The average integral signals of acetyl-CoA for ATCC 31280, HQG-3 and HQG-7 were 11,783, 14,181 and 22,313, respectively.

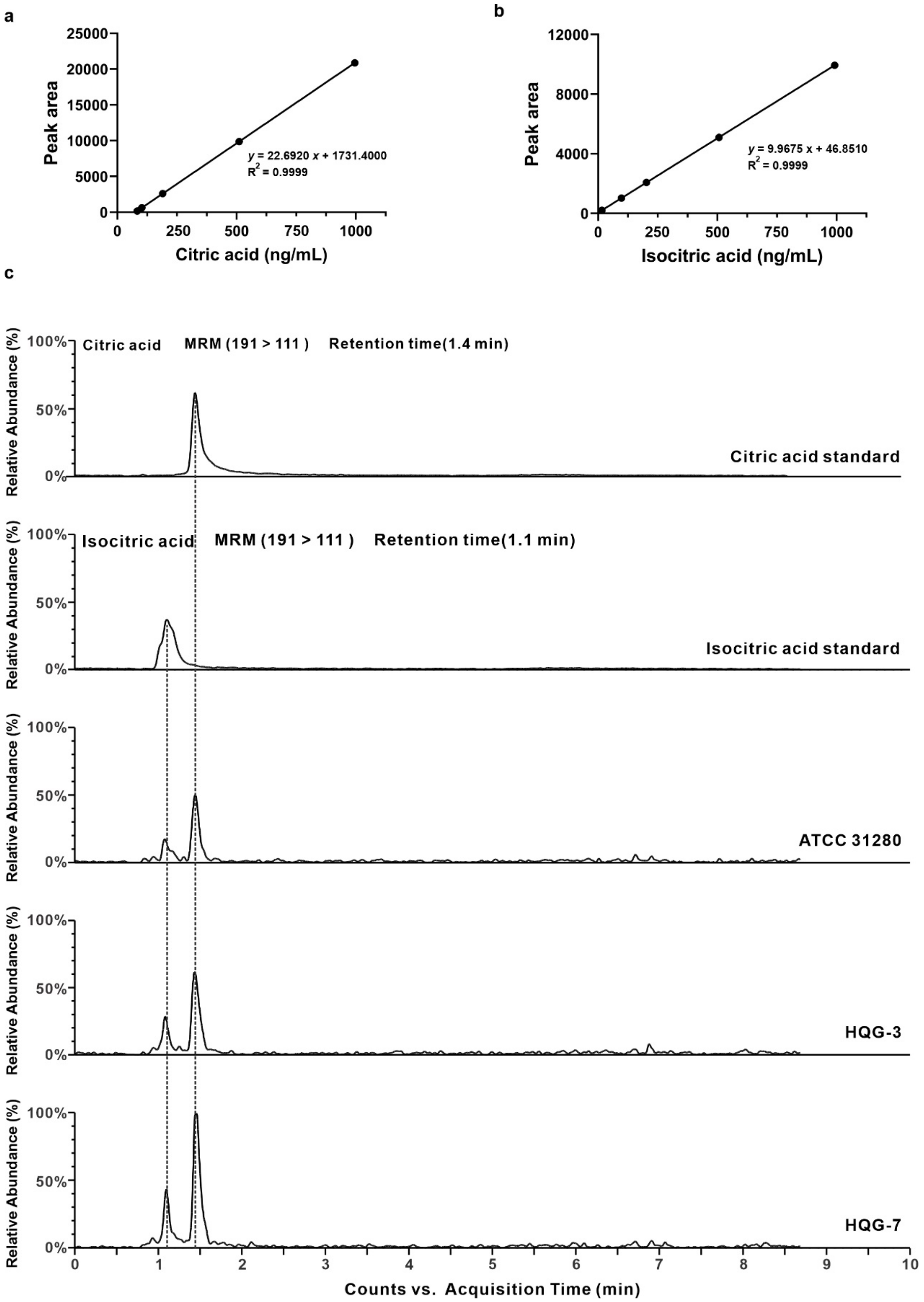

Supplementary Figure 27. Quantitative analysis of citric acid and isocitric acid.

a, Standard curve for citric acid. Calibration equation: $y = 22.692\ x + 1731.4$, $R^2 = 0.9999$. The citric acid concentrations used to generate the curve were 83, 103, 191, 510 and 996 ng/mL. **b,** Standard curve for isocitric acid. Calibration equation: $y = 9.9675\ x + 46.851$, R^2

= 0.9999. The isocitric acid concentrations used to generate the curve were 16, 98, 203, 507 and 992 ng/mL. **c,** MRM quantitative signals of compounds citric acid and isocitric acid in the wild-type ATCC 31280, HQG-3 and HQG-7. Precursor ion and qualitative ion (*m*/*z*) in MRM mode of citric acid and isocitric acid were both 191>111. The retention times of citric acid and isocitric acid were 1.40 min and 1.10 min, respectively. The average integral signals of citric acid for ATCC 31280, HQG-3 and HQG-7 were 7,301, 7,740 and 11,740, respectively, and the average integral signals of isocitric acid for ATCC 31280, HQG-3 and HQG-7 were 2,081, 2,792 and 4,482, respectively.

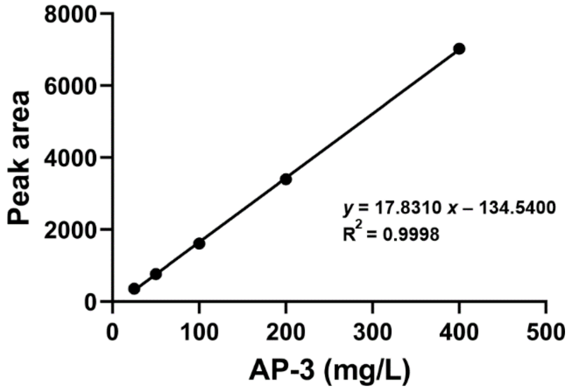

Supplementary Figure 28. Standard curve for AP-3.

Calibration equation: y = 17.831 x – 134.54, R^2 = 0.9998. The AP-3 concentrations used to prepare the curve were 25, 50, 100, 200 and 400 mg/L.

SUPPLEMENTARY REFERENCES

1. Paget, M.S.B., Chamberlin, L., Atrih, A., Foster, S.J. & Buttner, M.J. Evidence that the extracytoplasmic function sigma factor sigma^{E} is required for normal cell wall structure in *Streptomyces coelicolor* A3(2). *J. Bacteriol.* **181**, 204-211 (1999).
2. Wang, X.R., Wang, R.F., Kang, Q.J. & Bai, L.Q. The antitumor agent ansamitocin P-3 binds to cell division protein FtsZ in *Actinosynnema pretiosum*. *Biomolecules* **10**, (2020).
3. He, Y.L. et al. Two pHZ1358 derivative vectors for efficient gene knockout in *Streptomyces*. *J. Microbiol. Biotechnol.* **20**, 678-682 (2010).
4. Ha, H.S., Hwang, Y.I. & Choi, S.U. Application of conjugation using ØC31 *att*/*int* system for *Actinoplanes teichomyceticus*, a producer of teicoplanin. *Biotechnol. Lett.* **30**, 1233-1238 (2008).
