# Supplementary material for: Biosynthesis of ansamitocin P-3 incurs stress on the producing strain Actinosynnema pretiosum at multiple targets
Source: Commun Biol. 2023 Aug 18;6:860. doi: 10.1038/s42003-023-05227-w (PMC10439133; doi:10.1038/s42003-023-05227-w)
Supplement: Supplementary file 3 — Description of Additional Supplementary Files [file 42003_2023_5227_MOESM3_ESM.pdf]

Description of Additional Supplementary Files

File Name: Supplementary Data 1
Description: Source data underlying the graphs in this study following with exact p-values and Cohen's d values.

File Name: Supplementary Data 2
Description: Processed proteomic data used in this study.
